# Nutritional risk score drives postoperative morbidity in colorectal cancer: findings from a LASSO-selected multivariable model in 1013 consecutive patients

**DOI:** 10.3389/fnut.2026.1854727

**Published:** 2026-07-22

**Authors:** Lu Wang, Shun Wang, Liqin Deng

**Affiliations:** 1 Department of Anesthesiology and Perioperative Medicine, General Hospital of Ningxia Medical University, Yinchuan, Ningxia, China; 2 Department of Anesthesiology, Clinical Medical College and The First Affiliated Hospital of Chengdu Medical College, Chengdu, Sichuan, China

**Keywords:** colorectal cancer, LASSO regression, nomogram, nutritional risk screening, postoperative complications

## Abstract

**Background:**

Postoperative complications afflict approximately 40% of patients undergoing colorectal cancer (CRC) surgery and adversely affect long-term oncological outcomes. Reliable perioperative risk stratification tools that are both clinically interpretable and actionable remain limited.

**Methods:**

This retrospective cohort study enrolled 1,013 consecutive patients undergoing elective radical resection for histopathologically confirmed CRC at a single tertiary center. A nine-system composite complication endpoint was defined. Missing data were handled via multiple imputation by chained equations (MICE; *m* = 5). Candidate predictors were screened by univariable logistic regression and LASSO regularization; final predictors were entered into multivariable logistic regression with Rubin’s rules pooling. Model performance was assessed by AUC, bootstrap internal validation (1,000 iterations), Hosmer–Lemeshow calibration, and decision curve analysis (DCA). A nomogram was constructed for individualized risk estimation.

**Results:**

Of 1,013 patients, 372 (36.7%) experienced ≥1 postoperative complication. LASSO selected eight independent predictors: NRS-2002 nutritional risk score (aOR 1.502, 95% CI 1.312–1.720), laparoscopic approach (aOR 0.457, 95% CI 0.297–0.702), intraoperative blood loss (aOR 1.002/mL), right colon tumor location (aOR 1.502), NLR (aOR 1.080/unit), operative time (aOR 1.002/min), hemoglobin, and total bilirubin. The model achieved an apparent AUC of 0.689 (bootstrap-corrected 0.680), satisfactory calibration (Hosmer–Lemeshow *p* = 0.709), and net clinical benefit across threshold probabilities of 10–90% on DCA. Sensitivity analyses confirmed robustness across four pre-specified scenarios (AUC range 0.666–0.715).

**Conclusion:**

This LASSO-derived nomogram provides transparent, bedside-applicable risk stratification for postoperative composite complications in CRC surgery, identifying nutritional status as the dominant modifiable predictor and supporting targeted perioperative optimization.

## Introduction

Colorectal cancer (CRC) ranks as the third most commonly diagnosed malignancy and the second leading cause of cancer-related death worldwide. According to GLOBOCAN 2022, approximately 1.93 million incident cases and 904,000 deaths were recorded globally, accounting for 9.6% of all new cancer diagnoses (1). Demographic projections anticipate that annual incidence will reach 3.2 million by 2040, with the steepest proportional increases expected in low and middle-income countries undergoing rapid epidemiological transition (2). Of particular concern is the documented rise in early-onset CRC (age ≤50 years) across high-income nations—a trend driven by dietary and lifestyle shifts that conventional age-stratified screening programmes, designed for older cohorts, are poorly positioned to address (3). For patients presenting with resectable disease, improving surgical outcomes represents the most tangible near-term opportunity to reduce the population-level burden of CRC-related mortality.

Surgical resection remains the cornerstone of curative-intent treatment for localised and locoregional CRC (4). Yet despite meaningful advances in minimally invasive techniques and the broad adoption of Enhanced Recovery After Surgery (ERAS) protocols, postoperative complications continue to affect approximately 40% of patients undergoing elective colorectal resection, spanning a spectrum that includes anastomotic leakage, surgical site infection, thromboembolic events, and cardiopulmonary dysfunction (5–7). The consequences extend well beyond the immediate perioperative window: a systematic review and meta-analysis of 16 studies encompassing 37,192 patients demonstrated that complications following curative-intent resection significantly worsened overall survival (HR 1.36, 95% CI 1.15–1.61), disease-free survival (HR 1.41, 95% CI 1.11–1.80), and overall recurrence rate (8). Population-based registry data further confirm that anastomotic leakage independently confers excess cancer-specific mortality in stage II–III disease, illustrating how discrete perioperative events can exert lasting oncological consequences (9).

Reliable perioperative risk stratification is therefore a prerequisite for directing intensive ERAS pathways, nutritional prehabilitation, and heightened clinical surveillance towards patients who stand to benefit most. Classical instruments such as POSSUM and CR-POSSUM require 18 physiological and operative variables—many unavailable at the preoperative visit—and were primarily optimised for mortality prediction rather than multisystem morbidity risk. A landmark clinical implementation study developed and prospectively deployed an artificial-intelligence model trained on 18,403 CRC patients from Danish national registries, achieving an AUC of 0.79 for one-year mortality and demonstrating reduced postoperative complications when personalised, risk-stratified intervention bundles were applied (10). Whilst that model achieves superior discrimination through a substantially larger feature space, its machine-learning architecture limits direct clinical interpretability and bedside applicability without specialist computational infrastructure. Nomogram-based prediction models, constructed on penalised logistic regression with formal calibration and decision curve analysis (DCA), offer a transparent and immediately actionable alternative.

Among the perioperative determinants most amenable to preoperative optimisation, nutritional status has attracted sustained clinical interest. The Nutritional Risk Screening 2002 (NRS-2002)—endorsed by the European Society for Clinical Nutrition and Metabolism (ESPEN) and incorporated into contemporary ERAS guidelines—provides a validated, composite bedside tool integrating weight loss, reduced oral intake, disease severity, and patient age (6). A multinational randomised controlled trial demonstrated that multimodal prehabilitation incorporating nutritional supplementation significantly reduced postoperative medical complications (OR 0.48, 95% CI 0.26–0.89) in patients undergoing elective colorectal cancer surgery (11), establishing nutritional risk as an actionable rather than merely observational predictor. Concurrently, the neutrophil-to-lymphocyte ratio (NLR)—readily derivable from routine preoperative full blood count—has been established as a reliable surrogate of systemic inflammatory burden, independently associated with perioperative morbidity across multiple abdominal oncological resections (12). Intraoperative blood loss has similarly been confirmed through meta-analytic evidence as a dose-dependent determinant of both short-term complication rates and long-term survival following CRC surgery (13).

The integration of these multidimensional predictors within a regularised, internally validated nomogram—calibrated against a multi-system composite complication endpoint and evaluated for clinical utility via DCA—has not previously been reported for an unselected consecutive CRC surgical cohort. Against this background, the present study sought to develop and internally validate a LASSO-based nomogram for predicting postoperative composite complications—defined across nine organ-system domains—in 1013 consecutive patients undergoing CRC surgery at a single tertiary centre. Employing multiple imputation by chained equations for missing data, LASSO regularisation applied across all five imputed datasets, multivariable logistic regression with Rubin’s rules pooling, and bootstrap internal validation, we aimed to construct a parsimonious, clinically interpretable prediction tool that quantifies individual risk and identifies actionable perioperative targets (14, 15).

## Methods

### Study design and participants

This retrospective cohort study drew on consecutive medical records from the General Hospital of Ningxia Medical University. The research protocol was approved by the Ethics Committee of Ningxia Medical University General Hospital (approval number KYLL-2021-1045). Patients were eligible if they had a histopathologically confirmed colorectal adenocarcinoma and underwent elective radical resection under general anaesthesia. We excluded those who had emergency surgery, a concurrent primary malignancy, pre-existing organ dysfunction meeting any of our outcome definitions at admission, or age below 18 years. A total of 1,013 patients formed the analytic cohort and were stratified by whether any postoperative complication occurred. All research procedures were conducted in accordance with the ethical principles of the Declaration of Helsinki and adhered to relevant guidelines and regulations (16).

### Data collection

Sixty-four variables were extracted from structured electronic records and operative notes across six domains: demographics and anthropometrics; comorbidities and medical history (including Charlson Comorbidity Index and modified Frailty Index); preoperative risk scores (NRS-2002, ASA physical status); preoperative laboratory values [including haemoglobin, neutrophil-to-lymphocyte ratio (NLR), albumin, fibrinogen, and D-dimer]; cardiopulmonary function (echocardiographic ejection fraction, spirometry indices, and arterial blood gas parameters); and tumour and operative characteristics (including surgical approach, operative time, and intraoperative blood loss).

### Outcome definition

The primary outcome was a composite of postoperative complications, defined as the occurrence of at least one event across nine organ-system categories: hypoalbuminaemia; respiratory complications; thromboembolic events; infectious complications; postoperative nausea and vomiting (PONV); cardiac complications; anastomosis-related complications; gastrointestinal dysfunction; and hepatorenal insufficiency. Secondary outcomes comprised the individual incidence of each complication category, time to first flatus, time to first defaecation, total hospital stay, and 30-day all-cause mortality. Overall missing data were limited (3.3%). Little’s MCAR test indicated a missing-at-random mechanism, precluding complete-case analysis as the primary approach. Variables with fewer than 5% missing values were imputed using the median or mode; those with 5–20% missingness underwent multiple imputation by chained equations (MICE; *m* = 5 datasets, 10 iterations), with predictive mean matching, logistic regression, or proportional-odds models applied according to variable type.

### Statistical analysis

Prior to modelling, restricted cubic splines (4 knots) were fitted to all continuous predictors to assess linearity; seven variables exhibiting significant non-linearity (*p* < 0.05) were log(1 + x)-transformed in all subsequent analyses. Univariable logistic regression was performed for each candidate predictor; variables with *p* < 0.10 advanced to collinearity screening using variance inflation factors (VIF > 5 as the alert threshold) and a Spearman correlation matrix. Surviving variables then entered LASSO logistic regression (10-fold cross-validation, λ1se criterion) applied independently across all five imputed datasets; a variable was retained if its coefficient was non-zero in at least three of five datasets (majority-vote rule), yielding eight predictors.

The eight selected variables were entered simultaneously into a multivariable logistic regression model fitted across all five imputed datasets, with estimates pooled using Rubin’s rules. To verify the joint contribution of variables with non-significant individual Wald estimates, we compared the full model against a reduced model excluding hemoglobin and total bilirubin using a pooled likelihood ratio test (D3 statistic) across the five imputed datasets. Discrimination was assessed by the area under the ROC curve (AUC), with internal validation performed using 1,000 bootstrap iterations to estimate optimism-corrected AUC. Calibration was evaluated with the Hosmer–Lemeshow test and a Loess-smoothed calibration curve. Clinical utility was examined through decision curve analysis (DCA), comparing model net benefit against treat-all and treat-none reference strategies. A nomogram was constructed from the final model coefficients to facilitate individualised risk estimation at the bedside. Because the model incorporates intraoperative blood loss and operative time alongside preoperative and surgical-approach predictors, its complete score is intended to be calculated once these intraoperative parameters become available—typically at the completion of surgery—providing an updated risk estimate to inform postoperative management rather than a purely preoperative prediction.

Four pre-specified sensitivity analyses tested model robustness: redefining the composite outcome to exclude hypoalbuminaemia (SA1); restricting the sample to complete cases only (SA2); stratified modelling by surgical approach (SA3); and augmenting the base model with diffusing capacity of the lung for carbon monoxide in the available subset, with DeLong’s test used to compare AUCs (SA4). All analyses were performed in R version 4.5.2. Reporting follows STROBE and TRIPOD guidelines.

## Results

### Patient characteristics

**Figure 1:**
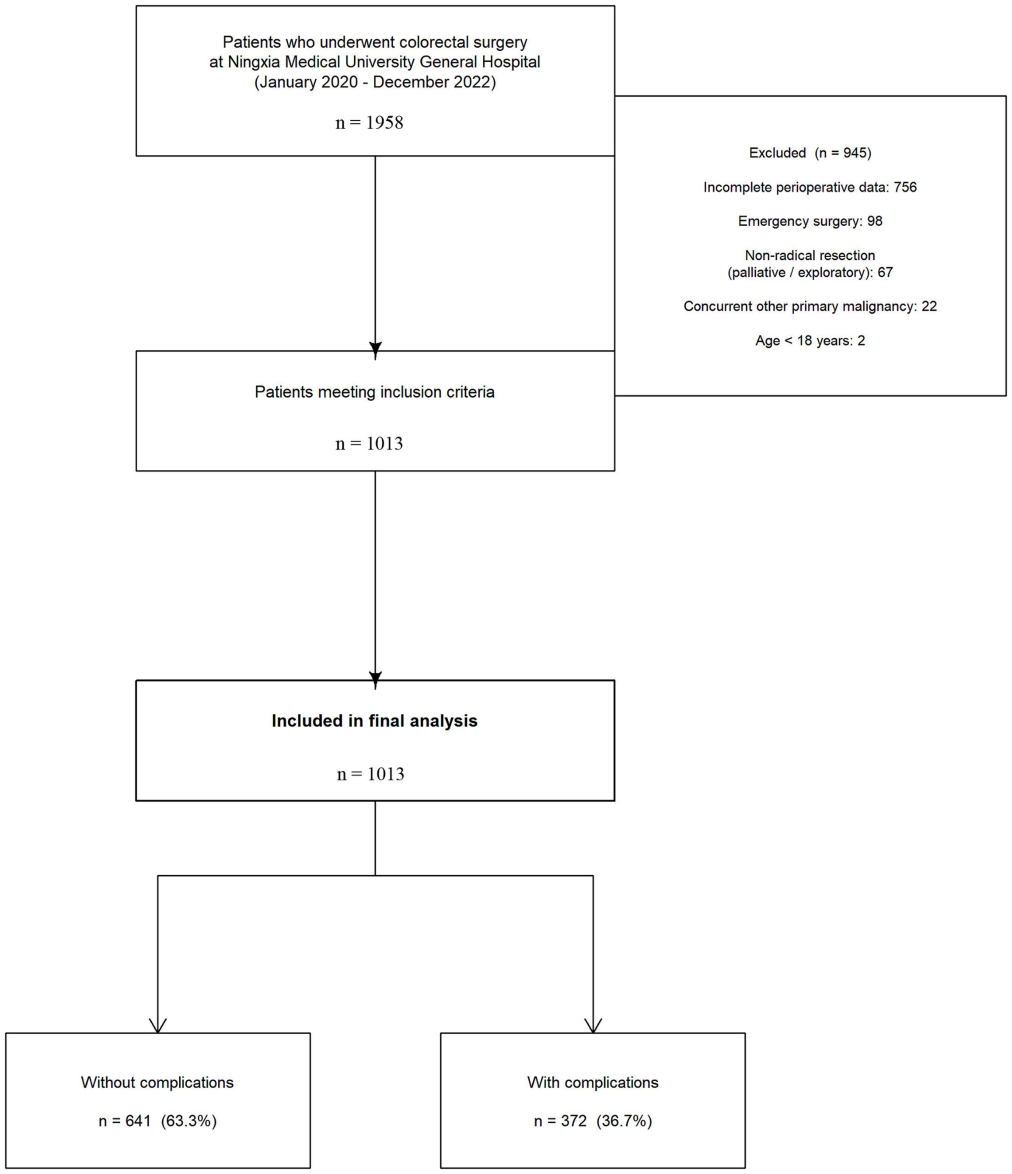
Flowchart of patient selection. Of 1958 patients screened, 945 were excluded (incomplete data, *n* = 756; emergency surgery, *n* = 98; non-radical resection, *n* = 67; concurrent malignancy, *n* = 22; age < 18 years, *n* = 2), yielding a final analytic cohort of 1,013 patients stratified by postoperative complication status.

Of the 1,013 patients enrolled, 372 (36.7%) developed one or more postoperative complications within the nine-system composite endpoint, whilst 641 (63.3%) remained complication-free (Figure 1). Thirty-day mortality was 1.1% (*n* = 11). Laparoscopic resection accounted for 88.5% of procedures (*n* = 897). Overall missing data were limited (3.3%), predominantly affecting pulmonary function indices and arterial blood gas parameters (9–10% each, Supplementary Figure S1); all variables with missingness exceeding 1% were handled via MICE (*m* = 5).

Between-group comparisons revealed that patients who experienced complications were older, had higher NLR, greater intraoperative blood loss, longer operative time, higher NRS-2002 scores, and more frequently underwent open surgery or presented with right colon tumours—all reaching statistical significance (*p* < 0.05). Preoperative haemoglobin was significantly lower in the complication group (median 125.0 vs. 134.0 g/L, SMD = 0.290). Full baseline characteristics and standardised mean differences are presented in Table 1.

**Table 1:** Baseline characteristics stratified by postoperative complication status.

Characteristic	Overall (*N* = 1,013)	No complication (*n* = 641)	Complication (*n* = 372)	*p* value	SMD
Demographics and general information					
Age (years), median (IQR)	64.00 (57.00–71.00)	64.00 (55.00–70.00)	67.00 (58.00–74.25)	<0.001	0.299
Sex, male, *n* (%)	600 (59.2%)	391 (61.0%)	209 (56.2%)	0.151	0.098
BMI (kg/m^2^), median (IQR)	23.53 (21.60–25.69)	23.67 (21.77–25.86)	23.44 (21.15–25.34)	0.058	0.025
Total hospital stay (days), median (IQR)	17.00 (14.00–21.00)	15.00 (13.00–19.00)	19.00 (16.00–24.00)	<0.001	0.690
Total hospitalization cost (CNY), median (IQR)	60590.14 (54546.13–68825.23)	57885.08 (52889.46–64123.46)	66141.61 (60408.44–76197.98)	<0.001	0.142
Comorbidities and medical history					
Hypertension, *n* (%)	328 (32.4%)	215 (33.5%)	113 (30.4%)	0.333	0.068
Coronary artery disease, *n* (%)	136 (13.4%)	69 (10.8%)	67 (18.0%)	0.002	0.208
Diabetes mellitus, *n* (%)	163 (16.1%)	112 (17.5%)	51 (13.7%)	0.138	0.104
Chronic lung disease, *n* (%)	13 (1.3%)	8 (1.2%)	5 (1.3%)	1.000	0.008
Smoking history, *n* (%)	171 (16.9%)	119 (18.6%)	52 (14.0%)	0.073	0.124
Alcohol use, *n* (%)	91 (9.0%)	67 (10.5%)	24 (6.5%)	0.042	0.144
Prior chemo/radiotherapy, *n* (%)	13 (1.3%)	8 (1.2%)	5 (1.3%)	1.000	0.008
Charlson comorbidity index, median (IQR)	0.00 (0.00–1.00)	0.00 (0.00–1.00)	0.00 (0.00–1.00)	0.745	0.070
Modified frailty index, *n* (%)				0.571	0.029
Mild	826 (81.5%)	523 (81.6%)	303 (81.5%)		
Moderate	173 (17.1%)	111 (17.3%)	62 (16.7%)		
Severe	14 (1.4%)	7 (1.1%)	7 (1.9%)		
NRS-2002 score, *n* (%)				<0.001	0.225
1	344 (34.0%)	242 (37.8%)	102 (27.4%)		
2	315 (31.1%)	229 (35.7%)	86 (23.1%)		
3	250 (24.7%)	131 (20.4%)	119 (32.0%)		
4	85 (8.4%)	34 (5.3%)	51 (13.7%)		
5	16 (1.6%)	4 (0.6%)	12 (3.2%)		
6	2 (0.2%)	0 (0.0%)	2 (0.5%)		
Preoperative risk scores					
ASA physical status, *n* (%)				0.006	0.129
I	1 (0.1%)	1 (0.2%)	0 (0.0%)		
II	531 (52.4%)	356 (55.5%)	175 (47.0%)		
III	470 (46.4%)	281 (43.8%)	189 (50.8%)		
IV	11 (1.1%)	3 (0.5%)	8 (2.2%)		
Hemoglobin (g/L), median (IQR)	131.00 (113.00–145.00)	134.00 (117.00–147.00)	125.00 (108.00–142.00)	<0.001	0.290
Preoperative laboratory values					
White blood cell (×10^9^/L), median (IQR)	5.82 (4.83–6.98)	5.89 (4.83–6.91)	5.79 (4.83–7.16)	0.754	0.152
Neutrophil count (×10^9^/L), median (IQR)	3.36 (2.60–4.32)	3.29 (2.59–4.20)	3.42 (2.64–4.58)	0.132	0.123
Lymphocyte count (×10^9^/L), median (IQR)	1.68 (1.28–2.10)	1.73 (1.34–2.13)	1.56 (1.20–2.03)	0.001	0.163
Neutrophil-to-lymphocyte ratio, median (IQR)	2.01 (1.43–2.92)	1.92 (1.43–2.77)	2.19 (1.45–3.28)	0.002	0.271
Albumin (g/L), median (IQR)	37.40 (34.77–40.49)	37.90 (35.36–40.91)	36.83 (33.90–39.40)	<0.001	0.315
Total protein (g/L), median (IQR)	64.50 (60.58–68.90)	64.80 (60.95–68.90)	63.65 (60.20–68.90)	0.030	0.153
ALT (U/L), median (IQR)	18.30 (12.30–25.60)	18.40 (12.60–25.10)	17.90 (11.75–26.00)	0.569	0.052
AST (U/L), median (IQR)	18.20 (14.90–23.30)	18.10 (15.10–23.10)	18.50 (14.50–23.42)	0.992	0.026
Total bilirubin (μmol/L), median (IQR)	10.83 (8.22–14.90)	11.20 (8.50–15.30)	10.17 (7.77–14.00)	0.006	0.137
Creatinine (μmol/L), median (IQR)	63.30 (54.40–74.00)	63.30 (55.10–73.60)	63.35 (53.80–75.12)	0.857	0.011
Blood urea nitrogen (mmol/L), median (IQR)	5.14 (4.12–6.17)	5.08 (4.09–6.06)	5.21 (4.21–6.41)	0.076	0.071
Fibrinogen (g/L), median (IQR)	3.04 (2.60–3.57)	2.93 (2.58–3.50)	3.17 (2.63–3.69)	0.002	0.117
D-dimer (mg/L), median (IQR)	0.47 (0.26–1.13)	0.40 (0.24–0.97)	0.59 (0.34–1.44)	<0.001	0.220
Ejection fraction (%), median (IQR)	66.91 (64.36–69.93)	66.92 (64.73–69.93)	66.91 (64.04–69.96)	0.516	0.056
Cardiopulmonary function					
CAD severity grade, *n* (%)				0.106	0.078
Grade 0	846 (83.5%)	548 (85.5%)	298 (80.1%)		
Grade 1	37 (3.7%)	20 (3.1%)	17 (4.6%)		
Grade 2	47 (4.6%)	27 (4.2%)	20 (5.4%)		
Grade 3	44 (4.3%)	20 (3.1%)	24 (6.5%)		
Grade 4	33 (3.3%)	22 (3.4%)	11 (3.0%)		
Grade 5	6 (0.6%)	4 (0.6%)	2 (0.5%)		
FVC (% predicted), median (IQR)	110.00 (99.00–122.00)	110.50 (101.00–122.00)	110.00 (97.75–122.00)	0.348	0.051
FEV₁ (% predicted), median (IQR)	103.00 (92.00–117.00)	104.00 (94.00–117.00)	102.00 (89.00–116.00)	0.018	0.155
FEV₁/FVC (%), median (IQR)	76.00 (71.00–80.07)	76.00 (71.00–80.37)	75.00 (69.00–80.00)	0.015	0.170
Arterial pH, median (IQR)	7.40 (7.39–7.42)	7.40 (7.39–7.42)	7.41 (7.39–7.42)	0.256	0.002
PaO₂ (mmHg), median (IQR)	70.20 (65.10–75.30)	70.60 (65.60–75.70)	69.80 (63.45–75.05)	0.010	0.024
PaCO₂ (mmHg), median (IQR)	38.40 (35.90–40.80)	38.50 (36.20–40.70)	38.30 (35.60–40.85)	0.249	0.087
Tumor and surgical characteristics					
Tumor location, *n* (%)				<0.001	0.186
Right colon	172 (17.0%)	84 (13.1%)	88 (23.7%)		
Left colon	182 (18.0%)	112 (17.5%)	70 (18.8%)		
Rectum	659 (65.1%)	445 (69.4%)	214 (57.5%)		
TNM stage, *n* (%)				0.191	0.087
I–II	581 (57.4%)	381 (59.4%)	200 (53.8%)		
III	356 (35.1%)	214 (33.4%)	142 (38.2%)		
IV	74 (7.3%)	44 (6.9%)	30 (8.1%)		
Tumor size, *n* (%)				0.023	0.130
<5 cm	576 (56.9%)	380 (59.3%)	196 (52.7%)		
≥5 cm	401 (39.6%)	234 (36.5%)	167 (44.9%)		
Unknown	34 (3.4%)	25 (3.9%)	9 (2.4%)		
Pathological differentiation, *n* (%)				0.074	0.078
Poor	78 (7.7%)	40 (6.2%)	38 (10.2%)		
Moderate	706 (69.7%)	452 (70.5%)	254 (68.3%)		
Well	227 (22.4%)	147 (22.9%)	80 (21.5%)		
Surgical approach, *n* (%)				<0.001	0.299
Laparoscopic	897 (88.5%)	590 (92.0%)	307 (82.5%)		
Open	114 (11.3%)	49 (7.6%)	65 (17.5%)		
Anesthesia type, *n* (%)				0.668	0.034
TIVA	872 (86.1%)	549 (85.6%)	323 (86.8%)		
Inhalation combined	141 (13.9%)	92 (14.4%)	49 (13.2%)		
Operative time (min), median (IQR)	218.00 (185.00–264.00)	215.00 (180.00–256.00)	223.50 (192.00–270.25)	0.001	0.197
Intraoperative blood loss (mL), median (IQR)	100.00 (50.00–100.00)	100.00 (50.00–100.00)	100.00 (80.00–200.00)	<0.001	0.356
Intraoperative RBC transfusion, *n* (%)	81 (8.0%)	37 (5.8%)	44 (11.8%)	<0.001	0.215
Stoma creation, *n* (%)	319 (31.5%)	203 (31.7%)	116 (31.2%)	0.928	0.010
Postoperative analgesia, *n* (%)	741 (73.1%)	482 (75.2%)	259 (69.6%)	0.064	0.125

Continuous variables: median (IQR), compared by Mann–Whitney U test. Categorical variables: *n* (%), compared by chi-square or Fisher’s exact test. SMD > 0.1 indicates a meaningful between-group difference. IQR, interquartile range; BMI, body mass index; NRS-2002, Nutritional Risk Screening 2002; ASA, American Society of Anesthesiologists; NLR, neutrophil-to-lymphocyte ratio; FVC, forced vital capacity; FEV₁, forced expiratory volume in one second; TIVA, total intravenous anaesthesia; RBC, red blood cell; SMD, standardised mean difference.

### Incidence of individual complication systems

Hypoalbuminaemia dominated the complication profile, occurring in 260 patients (25.7%). Other system-specific complications were less frequent: thromboembolic events 6.0% (*n* = 61), respiratory complications 5.5% (*n* = 56), PONV 4.2% (*n* = 43), infectious complications 3.8% (*n* = 39), cardiac complications 2.3% (*n* = 23), gastrointestinal dysfunction 2.3% (*n* = 23), anastomosis-related complications 2.2% (*n* = 22), and hepatorenal insufficiency 1.1% (*n* = 11). Full details of individual complication system prevalence are presented in Supplementary Table S1.

### Univariable analysis and nonlinearity assessment

Univariable logistic regression identified 27 variables meeting the *p* < 0.10 threshold for advancement to collinearity screening; one (log-transformed neutrophil count) was subsequently excluded for redundancy with NLR, leaving 26 variables that entered LASSO selection. Full results are presented in Table 2. Among continuous predictors explored via restricted cubic splines, NRS-2002 score exhibited a significant nonlinear dose–response relationship with complication risk (P-nonlinearity = 0.002), with risk accelerating markedly at scores ≥3. D-dimer demonstrated a sharp early rise plateauing at higher concentrations (P-nonlinearity = 0.002), and FEV₁/FVC showed a U-shaped pattern (P-nonlinearity = 0.011). In contrast, NLR and haemoglobin were adequately approximated by linear terms (P-nonlinearity > 0.05 for both). Spline curves for all continuous predictors are presented in Supplementary Figure S2.

**Table 2:** Univariable logistic regression of candidate predictors.

Variable	OR (95% CI)	*p*-value	Sig	Enter multivariable
Age (years)	1.029 (1.016–1.042)	<0.001	*	Yes
BMI (kg/m^2^)	1.006 (0.988–1.025)	0.487		No
CCI score	1.047 (0.962–1.140)	0.288		No
Hemoglobin (g/L)	0.987 (0.982–0.993)	<0.001	*	Yes
Lymphocyte count (×10^9^/L)	0.728 (0.592–0.896)	0.003	*	Yes
NLR	1.123 (1.058–1.193)	<0.001	*	Yes
Albumin (g/L)	0.934 (0.908–0.962)	<0.001	*	Yes
Total protein (g/L)	0.976 (0.956–0.996)	0.020	*	Yes
ALT (U/L)	1.001 (0.998–1.005)	0.467		No
AST (U/L)	0.997 (0.986–1.009)	0.647		No
Total bilirubin (μmol/L)	0.979 (0.957–1.002)	0.067	†	Yes
Creatinine (μmol/L)	1.000 (0.993–1.008)	0.909		No
Ejection fraction (%)	0.990 (0.971–1.010)	0.341		No
FVC (% predicted)	0.998 (0.990–1.005)	0.562		No
FEV₁ (% predicted)	0.994 (0.987–1.000)	0.060	†	Yes
PaO₂ (mmHg)	0.999 (0.995–1.003)	0.644		No
PaCO₂ (mmHg)	0.982 (0.950–1.016)	0.304		No
Arterial pH (per 0.1 unit)	1.326 (0.600–2.927)	0.438		No
Operative time (min)	1.003 (1.001–1.005)	0.003	*	Yes
Intraoperative blood loss (mL)	1.003 (1.002–1.004)	<0.001	*	Yes
WBC [log] (×10^9^/L)	1.460 (0.898–2.373)	0.127		No
Neutrophil [log] (×10^9^/L)	1.557 (1.067–2.273)	0.022	*	Yes
BUN [log] (mmol/L)	1.481 (0.953–2.302)	0.080	†	Yes
Fibrinogen [log] (g/L)	2.274 (1.145–4.520)	0.019	*	Yes
D-dimer [log] (mg/L)	1.702 (1.305–2.221)	<0.001	*	Yes
FEV₁/FVC [log] (%)	0.516 (0.250–1.066)	0.074	†	Yes
Sex (Male vs. Female)	0.820 (0.632–1.063)	0.133		No
Hypertension	0.869 (0.659–1.145)	0.317		No
Coronary artery disease	1.821 (1.265–2.621)	0.001	*	Yes
Diabetes mellitus	0.750 (0.524–1.075)	0.117		No
Chronic lung disease	1.078 (0.350–3.324)	0.896		No
Smoking history	0.713 (0.500–1.016)	0.061	†	Yes
Alcohol use	0.597 (0.367–0.971)	0.038	*	Yes
Prior chemo/radiotherapy	1.078 (0.350–3.324)	0.896		No
Intraoperative RBC transfusion	2.051 (1.290–3.262)	0.002	*	Yes
Stoma creation	0.978 (0.742–1.288)	0.872		No
Postoperative analgesia	0.741 (0.556–0.987)	0.040	*	Yes
Laparoscopic approach (vs Open)	0.392 (0.264–0.584)	<0.001	*	Yes
NRS-2002 score (per 1 point)	1.576 (1.387–1.791)	<0.001	*	Yes
ASA grade (per 1 grade)	1.470 (1.151–1.879)	0.002	*	Yes
mFI grade (per 1 grade)	1.051 (0.783–1.410)	0.742		No
TNM stage (per 1 stage)	1.191 (0.974–1.457)	0.089	†	Yes
CAD severity grade (per 1 grade)	1.104 (0.981–1.244)	0.101		No
Tumor location	—	<0.001	*	Yes
Right colon vs. rectum	2.178 (1.549–3.064)	<0.001	*	
Left colon vs. rectum	1.300 (0.924–1.827)	0.131		
Tumor size	—	0.015	*	Yes
≥5 cm vs. < 5 cm	1.386 (1.065–1.803)	0.015	*	
Other vs. < 5 cm	0.698 (0.319–1.527)	0.368		
Pathological differentiation	—	0.027	*	Yes
Poor vs. moderate	1.702 (1.063–2.725)	0.027	*	
Well vs. moderate	0.972 (0.710–1.331)	0.861		
Anesthesia type (TIVA vs. inhalation)	—	0.601		No
TIVA vs. inhalation	1.105 (0.760–1.605)	0.601		

OR (95% CI) and *p*-value are reported for each variable. Variables with *p* < 0.10 advanced to LASSO selection. Continuous variables with significant non-linearity were log(1 + x)-transformed [denoted (log)]. OR, odds ratio; CI, confidence interval; NLR, neutrophil-to-lymphocyte ratio; [log], log(1 + x)-transformed. *p < 0.05; †*p* < 0.10.

### LASSO variable selection

**Figure 2:**
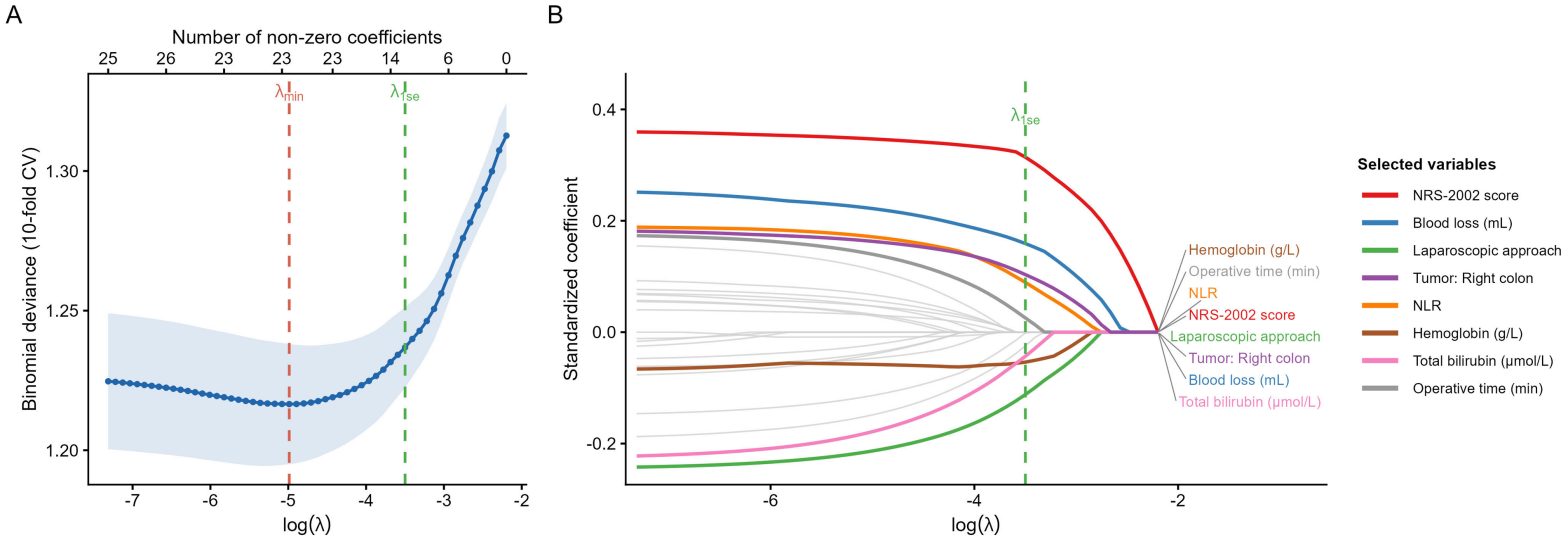
LASSO variable selection. **(A)** Ten-fold cross-validated binomial deviance versus log(*λ*). Red dashed line: λ ~ min~; green dashed line: λ ~ 1se ~ (selection criterion). Numbers on top indicate non-zero coefficients at each λ. **(B)** Standardised coefficient paths for all 26 candidate variables. Colored trajectories indicate the eight variables retained at λ ~ 1se ~ across ≥3 of 5 imputed datasets. LASSO, least absolute shrinkage and selection operator; NLR, neutrophil-to-lymphocyte ratio.

The coefficient path and cross-validation deviance curves, based on the 26 variables surviving collinearity screening, are presented in Figure 2. Inter-predictor Spearman correlations are provided in Supplementary Figure S3, confirming the absence of problematic collinearity among retained predictors. Selection frequencies for all 26 variables across the five imputed datasets are reported in Supplementary Table S3; two of the eight final predictors (total bilirubin, operative time) were retained by a narrow majority (3 of 5 datasets). An alternative analysis applying LASSO to the five datasets pooled into a single stacked dataset with case weights of 1/5 per imputation converged reliably and selected 20 of the 26 candidate variables; this larger set fully contained all eight predictors of the primary model, with no primary predictor excluded, supporting the robustness of the final variable selection.

### Multivariable logistic regression

The eight LASSO-selected variables were entered simultaneously into multivariable logistic regression (*N* = 1,013; events = 372, 36.7%; Nagelkerke *R^2^* = 0.154). NRS-2002 score emerged as the dominant independent predictor (aOR 1.502, 95% CI 1.312–1.720, *p* < 0.001). Laparoscopic approach conferred substantial protection against complications relative to open surgery (aOR 0.457, 95% CI 0.297–0.702, *p* < 0.001). Intraoperative blood loss (aOR 1.002 per mL, 95% CI 1.001–1.003, *p* = 0.001), right colon tumour location versus rectum (aOR 1.502, 95% CI 1.044–2.162, *p* = 0.028), NLR (aOR 1.080 per unit, 95% CI 1.015–1.150, *p* = 0.016), and operative time (aOR 1.002 per min, 95% CI 1.000–1.004, *p* = 0.021) each independently predicted the composite outcome. Haemoglobin and total bilirubin did not reach conventional significance (*p* = 0.243 and *p* = 0.080, respectively) but were retained on the basis of consistent LASSO selection across all five imputed datasets and their contribution to model calibration (Table 3). A pooled likelihood ratio test comparing the full model against a reduced model excluding these two variables confirmed their joint contribution was unlikely due to chance (*F*(2, 318.9) = 3.848, *p* = 0.022), with consistently better fit across all five imputed datasets (mean AIC 1227.8 ± 2.0 vs. 1232.2 ± 0.8; mean AUC 0.689 ± 0.002 vs. 0.682 ± 0.000).

**Table 3:** Multivariable logistic regression of independent predictors for postoperative composite complications.

Variable	*β*	SE	aOR	95% CI	*p*-value
NRS-2002 score	0.407	0.069	1.502	1.312–1.720	<0.001
Blood loss (mL)	0.002	0.001	1.002	1.001–1.003	0.001
Laparoscopic approach	−0.783	0.219	0.457	0.297–0.702	<0.001
Tumor: right colon	0.407	0.186	1.502	1.044–2.162	0.028
NLR	0.077	0.032	1.080	1.015–1.150	0.016
Hemoglobin (g/L)	−0.004	0.003	0.996	0.989–1.003	0.243
Total bilirubin (μmol/L)	−0.023	0.013	0.977	0.952–1.002	0.080
Operative time (min)	0.002	0.001	1.002	1.000–1.004	0.021
Model statistics					
Sample size (N)					1,013
Events, *n* (%)					372 (36.7%)
Nagelkerke *R*^2^					0.154
Hosmer-lemeshow *χ*^2^					5.448
Hosmer-lemeshow *P*					0.709

Eight LASSO-selected variables were modelled across five imputed datasets and pooled by Rubin’s rules. β, regression coefficient; SE, standard error; aOR, adjusted odds ratio; CI, confidence interval. Model statistics: *N* = 1,013; events = 372 (36.7%); Nagelkerke *R*^2^ = 0.154; Hosmer–Lemeshow *p* = 0.709.

### Model performance and clinical utility

**Figure 3:**
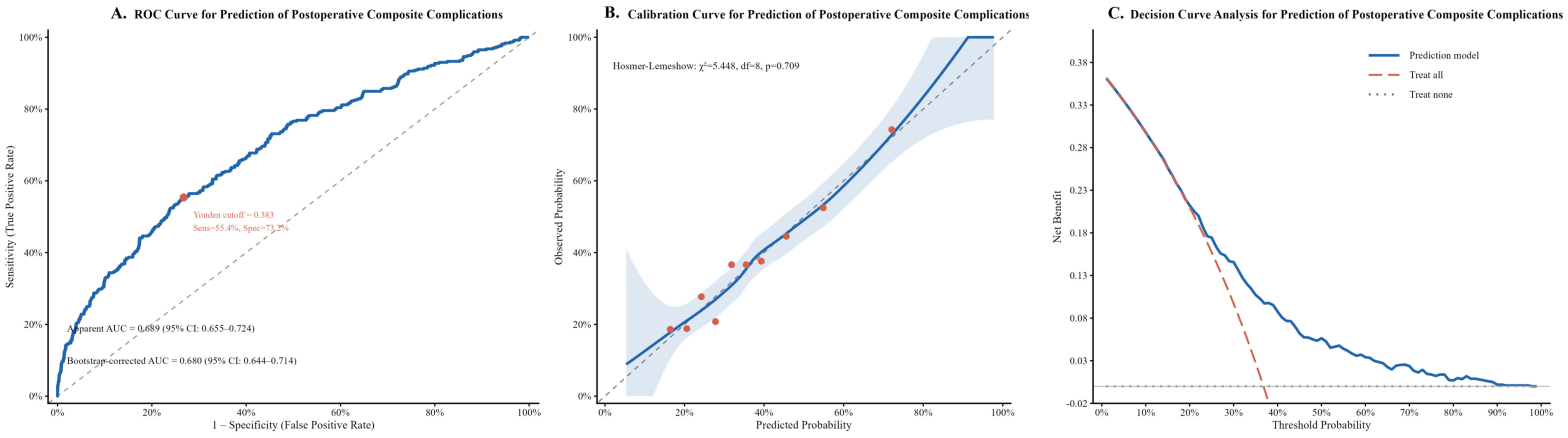
Discrimination, calibration, and clinical utility of the prediction model. **(A)** ROC curve. Apparent AUC = 0.689 (95% CI 0.655–0.724); bootstrap-corrected AUC = 0.680. Youden-optimal threshold = 0.383 (sensitivity 55.4%, specificity 73.2%). **(B)** Calibration curve. Loess-smoothed curve with 95% confidence band; Hosmer–Lemeshow *χ*^2^ = 5.448, df = 8, *p* = 0.709. **(C)** Decision curve analysis. The model provides net benefit over treat-all and treat-none strategies across threshold probabilities of 10–90%. AUC, area under the receiver operating characteristic curve; ROC, receiver operating characteristic.

**Figure 4:**
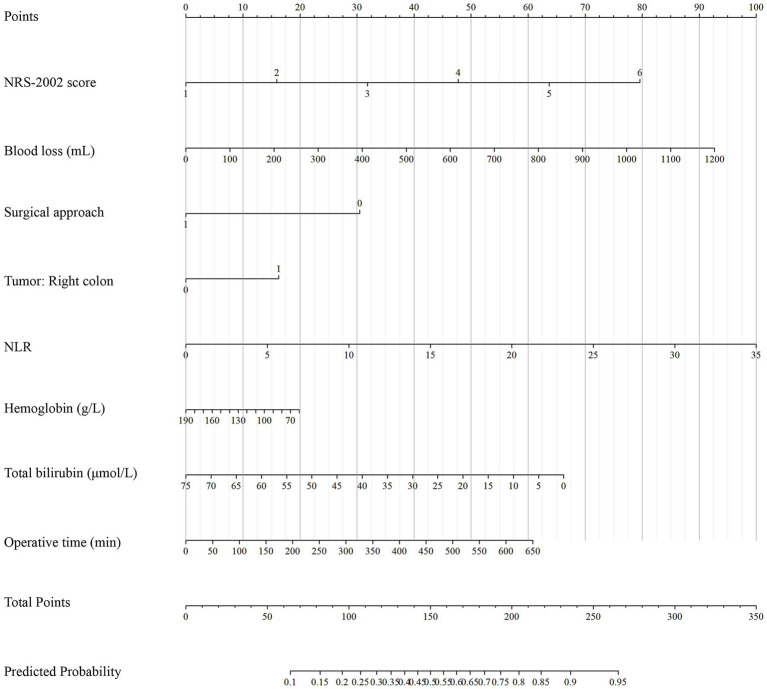
Nomogram for Prediction of Postoperative Composite Complications. Points for each predictor are summed to derive Total Points, from which the predicted complication probability is read on the bottom scale. NRS-2002, Nutritional Risk Screening 2002; NLR, neutrophil-to-lymphocyte ratio.

Discriminative ability, calibration, and clinical utility of the final model are presented in Figure 3, respectively. Apparent discriminative ability was moderate (AUC = 0.689, 95% CI 0.655–0.724); bootstrap-corrected AUC was 0.680, indicating minimal overfitting (optimism = 0.009). The Youden-optimal probability threshold was 0.383, with corresponding sensitivity of 55.4% and specificity of 73.2%. Calibration was satisfactory, with the Hosmer–Lemeshow test yielding *χ*^2^ = 5.448 (df = 8, *p* = 0.709), and the calibration curve demonstrating close concordance between predicted and observed probabilities across the full risk range. Decision curve analysis demonstrated net clinical benefit over both treat-all and treat-none reference strategies across threshold probabilities of approximately 10–90%. A nomogram translating the final model coefficients into individualised bedside risk estimates is presented in Figure 4. To estimate an individual patient’s risk, the value of each predictor is located on its corresponding axis and a vertical line drawn upward to the Points scale to obtain that variable’s score; the eight individual scores are summed to derive the Total Points, and a vertical line drawn downward from the Total Points axis to the bottom scale yields the predicted probability of composite postoperative complications, following the point-based reading convention used in previously published prognostic and risk-prediction nomograms (17, 18). Consistent with the timing clarified above, this calculation is intended to be performed once intraoperative blood loss and operative time are recorded, typically at the completion of surgery.

### Sensitivity analyses

Model robustness was confirmed across all four pre-specified scenarios (Supplementary Table S2). Redefining the composite outcome after excluding hypoalbuminaemia (SA1; event rate 18.1%) yielded AUC = 0.688, virtually identical to the main model (0.689). All eight predictors retained the same direction of association in the SA1 re-estimation, with modest shifts in magnitude for NLR and operative time, supporting the interpretation that the model’s associations are not specific to hypoalbuminaemia but reflect a broader perioperative risk signal. The proportion of outcome variance explained was nonetheless lower in SA1 (Nagelkerke *R*^2^ = 0.118 vs. 0.154 in the main model), reflecting hypoalbuminaemia’s substantial contribution to the original composite’s overall variability. Complete-case analysis (SA2; *n* = 813) produced nearly identical performance (AUC = 0.687), affirming that multiple imputation introduced no material bias. Within the laparoscopic subgroup (SA3; *n* = 899), AUC was 0.666; the open-surgery subgroup (*n* = 116, AUC = 0.803) remained exploratory given limited statistical power. Augmenting the base model with diffusing capacity of the lung for carbon monoxide in the available subset (SA4; *n* = 500) did not meaningfully improve discrimination (DeLong *p* = 0.677). Across all sensitivity scenarios, AUC ranged from 0.666 to 0.715 and Hosmer–Lemeshow p consistently exceeded 0.05, supporting the stability of the primary model.

### Secondary outcomes

Composite complications were independently associated with delayed time to first flatus (GMR 1.080, 95% CI 1.025–1.138, *p* = 0.004), delayed time to first stool (GMR 1.084, 95% CI 1.033–1.137, *p* = 0.001), and substantially prolonged operative length of stay (GMR 1.248, 95% CI 1.199–1.300, *p* < 0.001). Stoma creation was independently associated with earlier restoration of intestinal function (first flatus: GMR 0.853, *p* < 0.001; first stool: GMR 0.831, *p* < 0.001), whereas neither surgical approach nor TNM stage reached significance for any recovery endpoint after covariate adjustment. Full secondary outcome analyses are presented in Supplementary Table S1.

## Discussion

This study developed and internally validated a LASSO-based nomogram for predicting postoperative composite complications in 1013 consecutive patients undergoing colorectal cancer surgery. Across a nine-system composite endpoint, eight independent predictors were identified—with the NRS-2002 nutritional risk score as the dominant contributor—yielding moderate discrimination (AUC 0.689), robust calibration (Hosmer–Lemeshow *p* = 0.709), and net clinical benefit across decision curve analysis threshold probabilities of approximately 10–90%. Bootstrap internal validation confirmed minimal overfitting (optimism-corrected AUC 0.680), representing modest discrimination. At the Youden-optimal threshold (0.383), sensitivity was 55.4%—meaning 44.6% of patients who developed a complication would not have been flagged as high-risk—corresponding to a positive predictive value of 54.5% given the observed 36.7% complication rate. At this threshold, the model would flag 37.3 of every 100 patients as high-risk, a 62.6% reduction in interventions relative to treating all patients, but at the cost of missing 44.6% of complications—underscoring that any single operating point reflects a clinical trade-off between missed complications and unnecessary intervention, not one the model can resolve unaided. We therefore advise caution in interpreting the nomogram’s output at the individual level and do not recommend its use to rule complications in or out for a given patient. Its value instead lies in population-level risk stratification, identifying candidates for perioperative prehabilitation, anaemia correction, and enhanced postoperative surveillance, with the operating threshold chosen according to the relative costs of intervention and missed complications in a given setting.

NRS-2002 emerged as the strongest independent predictor (aOR 1.502, *p* < 0.001), with restricted cubic spline analysis confirming a nonlinear dose–response relationship steepening markedly above a score of 3. Nutritional compromise impairs immunocompetence, wound healing, and physiological reserve, collectively amplifying vulnerability to surgical stress. A multinational randomised controlled trial demonstrated that multimodal prehabilitation incorporating nutritional supplementation reduced postoperative medical complications by 52% in elective colorectal cancer surgery (OR 0.48, 95% CI 0.26–0.89) (11). Establishing nutritional risk as an actionable intervention target. A parallel community-based exercise prehabilitation randomised controlled trial corroborated this benefit among patients stratified by preoperative nutritional risk (19). Current ERAS Society guidelines accordingly mandate NRS-2002 screening and preoperative oral nutritional supplementation for at-risk patients (6).

Laparoscopic resection independently reduced composite complication odds by 54.3% relative to open surgery (aOR 0.457, 95% CI 0.297–0.702, *p* < 0.001). A meta-analysis of randomised controlled trials demonstrated significantly lower postoperative IL-6 and CRP levels following laparoscopic versus open colorectal resection, quantifying the attenuated systemic inflammatory response of the minimally invasive approach (20). A complementary systematic review and meta-analysis confirmed substantially lower IL-6 concentrations in laparoscopic cohorts at 3–9 h post-surgery, reinforcing the immunological rationale for the observed protective effect (21). The attenuation of model discrimination within the laparoscopic subgroup may partly reflect diminished outcome heterogeneity within this more homogeneous stratum, rather than an inherent limitation of the model itself. Conversely, the open-surgery subgroup—enriched for greater disease complexity and physiological heterogeneity by virtue of its selection for cases unsuitable for minimally invasive resection—demonstrated substantially superior discrimination, surpassing both the laparoscopic stratum and the overall cohort. Of particular potential relevance is that open colorectal resection disproportionately affects patients with advanced local disease or unfavourable anatomy, precisely those for whom accurate postoperative risk stratification is most consequential yet least supported by existing prediction models developed predominantly in laparoscopic cohorts. The superior discriminative performance observed in this stratum may partly reflect the greater variability in intraoperative blood loss and operative duration inherent to open procedures, lending the model’s surgical complexity surrogates enhanced prognostic leverage where it matters most. This finding should nonetheless be interpreted cautiously given the limited subgroup size and exploratory nature of the analysis; external validation in an adequately powered open-surgery cohort is warranted before approach-specific surveillance strategies can be recommended.

Both parameters serve as quantifiable surrogates of surgical complexity and haemostatic management. A systematic review and meta-analysis of 43 studies in 59,813 patients established that increased operative blood loss was independently associated with higher overall morbidity and adverse long-term oncological outcomes (13). A further meta-analysis confirmed that greater intraoperative blood loss was associated with a 2.06-fold higher complication risk and significantly worse overall survival (HR 1.45, 95% CI 1.17–1.80) (22). These findings substantiate both predictors as independent prognostic inputs beyond anatomical staging alone.

NLR independently predicted composite complications (aOR 1.080 per unit, *p* = 0.016). A large systematic review and meta-analysis of 100 studies in 40,559 patients found that elevated pretreatment NLR was independently associated with shorter overall survival across solid tumours (pooled HR 1.81, 95% CI 1.67–1.97) (12), driven by protumorigenic neutrophil activity and suppressed lymphocyte-mediated antitumour immunity. Consistent selection across all five imputed datasets validates its independent contribution despite modest per-unit effect magnitude.

Right colon tumour location conferred 50.2% higher complication odds versus rectal tumours (aOR 1.502, *p* = 0.028), reflecting technical complexity near the superior mesenteric vessels. A national audit of 29,274 right hemicolectomy patients demonstrated stable complication rates despite increasing laparoscopic adoption, underscoring an anatomically irreducible risk component (23). A prospective multicentre study further confirmed intraoperative blood loss and anastomotic configuration as independent morbidity predictors after right hemicolectomy (24). Hemoglobin and intraoperative blood loss—plausible correlates of right-sided tumour biology and occult blood loss—were entered alongside right colon location within the same multivariable model, so the reported effect already reflects adjustment for these factors. TNM stage met univariable screening (*p* = 0.089) but was not retained by LASSO, and was therefore not separately adjusted for. Molecular and histological features distinguishing right from left-sided tumours were not captured in our dataset and remain unmeasured. We interpret the effect as predominantly reflecting anatomical and technical complexity of right-sided resection rather than stage or biology per se, consistent with the cited evidence on right hemicolectomy as an anatomically distinct risk category (23, 24).

Haemoglobin and total bilirubin were retained on the basis of consistent LASSO selection and calibration contribution, consistent with the established association between preoperative anaemia and perioperative morbidity (25, 26).

Hypoalbuminaemia was the most prevalent component of the primary composite endpoint, accounting for 260 of 372 events (70.2%), raising the question of whether the model’s apparent performance was driven largely by this single complication. Reassuringly, excluding it from the composite (SA1) left the apparent AUC essentially unchanged (0.688 vs. 0.689), and coefficient comparison (Supplementary Table S2B) showed that all eight predictors retained the same direction of association, with five of eight remaining statistically significant and none reversing direction. Two predictors attenuated modestly—NLR (*p* = 0.016 to 0.279) and intraoperative blood loss (*p* = 0.001 to 0.028)—which may partly reflect overlapping signal with hypoalbuminaemia’s own inflammatory pathophysiology, in which albumin is redistributed into the interstitium via increased capillary permeability and undergoes accelerated catabolism (27). Operative time strengthened slightly (*p* = 0.021 to 0.007), consistent with its established role as a risk factor for anastomotic leakage and other structural complications via bacterial exposure and tissue ischaemia during prolonged procedures (28). NRS-2002 and laparoscopic approach remained robustly significant in both models. Nagelkerke *R*^2^ fell from 0.154 to 0.118 after exclusion, indicating hypoalbuminaemia accounted for a non-trivial share of the composite’s explanatory power despite not driving discrimination. Together, these findings support the multi-system composite endpoint and indicate the model’s performance does not hinge on a single high-prevalence component.

Classical POSSUM instruments require 18 variables—many unavailable preoperatively—and were optimised for mortality prediction. A landmark clinical implementation study deploying an artificial-intelligence model trained on 18,403 CRC patients achieved an AUC of 0.79 for one-year mortality and demonstrated reductions in postoperative complications through personalised risk-stratified intervention bundles (10). Whilst that model achieves superior discrimination through a substantially larger feature space, our nomogram offers transparency, interpretability, and immediate bedside applicability at the point of surgical completion, without computational infrastructure. A LASSO-derived prediction model for severe complications in elderly CRC patients reported an optimism-corrected AUC of 0.65 across five Dutch hospitals (29), against which our bootstrap-corrected AUC of 0.680 compares favourably despite a nine-system composite endpoint—a methodological strength that captures the full breadth of perioperative morbidity.

### Clinical implications

Decision curve analysis confirmed net clinical benefit across threshold probabilities of 10–90%. Among the eight predictors, NRS-2002 score and haemoglobin are directly actionable before surgery: multimodal prehabilitation incorporating nutritional supplementation reduces complication rates (OR 0.74, 95% CI 0.59–0.94) and shortens hospital stay (mean difference −2.47 days) (30), while preoperative anaemia correction offers a parallel target (25, 26). Surgical approach is a clinical decision rather than a model output, whereas blood loss and operative time reflect surgical complexity rather than preoperatively modifiable factors. Guideline-recommended nutritional supplementation, minimally invasive surgery, and goal-directed haemodynamic therapy align with these targets (31). The complete nomogram, calculated once intraoperative findings are known, instead informs postoperative decisions: composite complications independently prolonged time to flatus, stool, and hospital stay (GMR 1.080–1.248), underscoring the value of this updated estimate for guiding surveillance and discharge planning.

### Limitations and future directions

Several limitations warrant acknowledgement. First, the retrospective single-centre design introduces potential selection bias and constrains generalisability to institutions with differing case-mix profiles and ERAS adherence. Second, the absence of independent external validation remains the most consequential limitation; bootstrap optimism correction partially mitigates but does not substitute for true external validation, and reporting follows TRIPOD guidelines (32). Third, hypoalbuminaemia dominated the composite endpoint. Although discrimination and predictor direction were preserved after its exclusion, the corresponding reduction in explained variance noted above suggests the model’s predictive contribution is disproportionately weighted toward nutritionally-mediated risk, with more modest precision for structural complications independent of nutritional status. Fourth, the predominantly laparoscopic cohort (88.5%) limits predictor contrast within that stratum (AUC 0.666). PROBAST-guided risk-of-bias assessment identifies the single-centre retrospective design and absence of external validation as the primary methodological vulnerabilities (14). Future work should prioritise prospective multicentre external validation, and longitudinal interventional studies examining whether nomogram-guided stratification—particularly nutritional prehabilitation for NRS-2002 ≥ 3—translates into measurable reductions in composite morbidity would establish clinical impact beyond risk prediction alone.

## Conclusion

In a consecutive cohort of 1,013 patients undergoing elective colorectal cancer surgery, a LASSO-regularised multivariable nomogram incorporating eight preoperatively or intraoperatively available predictors achieved moderate discrimination, satisfactory calibration, and net clinical benefit across a broad range of decision thresholds. The NRS-2002 nutritional risk score was the dominant independent predictor, exhibiting a nonlinear dose–response relationship with composite complication risk that accelerates markedly at scores ≥3. Laparoscopic surgical approach, intraoperative blood loss, right colon tumour location, NLR, haemoglobin, total bilirubin, and operative time each contributed independently to risk stratification. NRS-2002 score and haemoglobin are amenable to preoperative optimisation through nutritional prehabilitation and anaemia correction, respectively, while surgical approach represents a further modifiable clinical decision; the complete nomogram, incorporating intraoperative blood loss and operative time once known, in turn provides an updated postoperative risk estimate to guide targeted surveillance. External multicentre validation and prospective interventional studies examining NRS-2002-guided prehabilitation and preoperative anaemia correction are warranted to establish the model’s broader clinical impact.

## Data Availability

The raw data supporting the conclusions of this article will be made available by the authors, without undue reservation.

## Supplementary material

SUPPLEMENTARY FIGURE S1 Missing data pattern across predictor variables (*N* = 1013). Black bars indicate missing values; gray indicates present data. Overall missingness was 3.3%, predominantly affecting total bilirubin (14%), pulmonary function indices (~10%), and arterial blood gas parameters (~9%). Variables with >1% missingness were handled by MICE (*m* = 5). MICE, multiple imputation by chained equations.

SUPPLEMENTARY FIGURE S2 Restricted cubic spline analyses of continuous predictors. Odds ratios (solid line) with 95% confidence intervals (shaded band) for each continuous predictor, referenced to its median. P~overall~: overall association; P~non-linearity~: departure from linearity. Variables with P~non-linearity~ < 0.05 were log(1+x)-transformed in subsequent analyses. OR, odds ratio; CI, confidence interval.

SUPPLEMENTARY FIGURE S3 Spearman correlation matrix of 27 candidate predictor variables. Color intensity reflects correlation magnitude (red: positive; blue: negative; scale: −1 to +1). All inter-predictor correlations were low (|*r*| < 0.50), confirming the absence of problematic collinearity. NLR, neutrophil-to-lymphocyte ratio.
