# Supplementary material for: Nutritional risk score drives postoperative morbidity in colorectal cancer: findings from a LASSO-selected multivariable model in 1013 consecutive patients
Source: Front Nutr. 2026 Jul 22;13:1854727. doi: 10.3389/fnut.2026.1854727 (PMC13437264; doi:10.3389/fnut.2026.1854727)
Supplement: Supplementary file 1 [file Image_1.pdf]

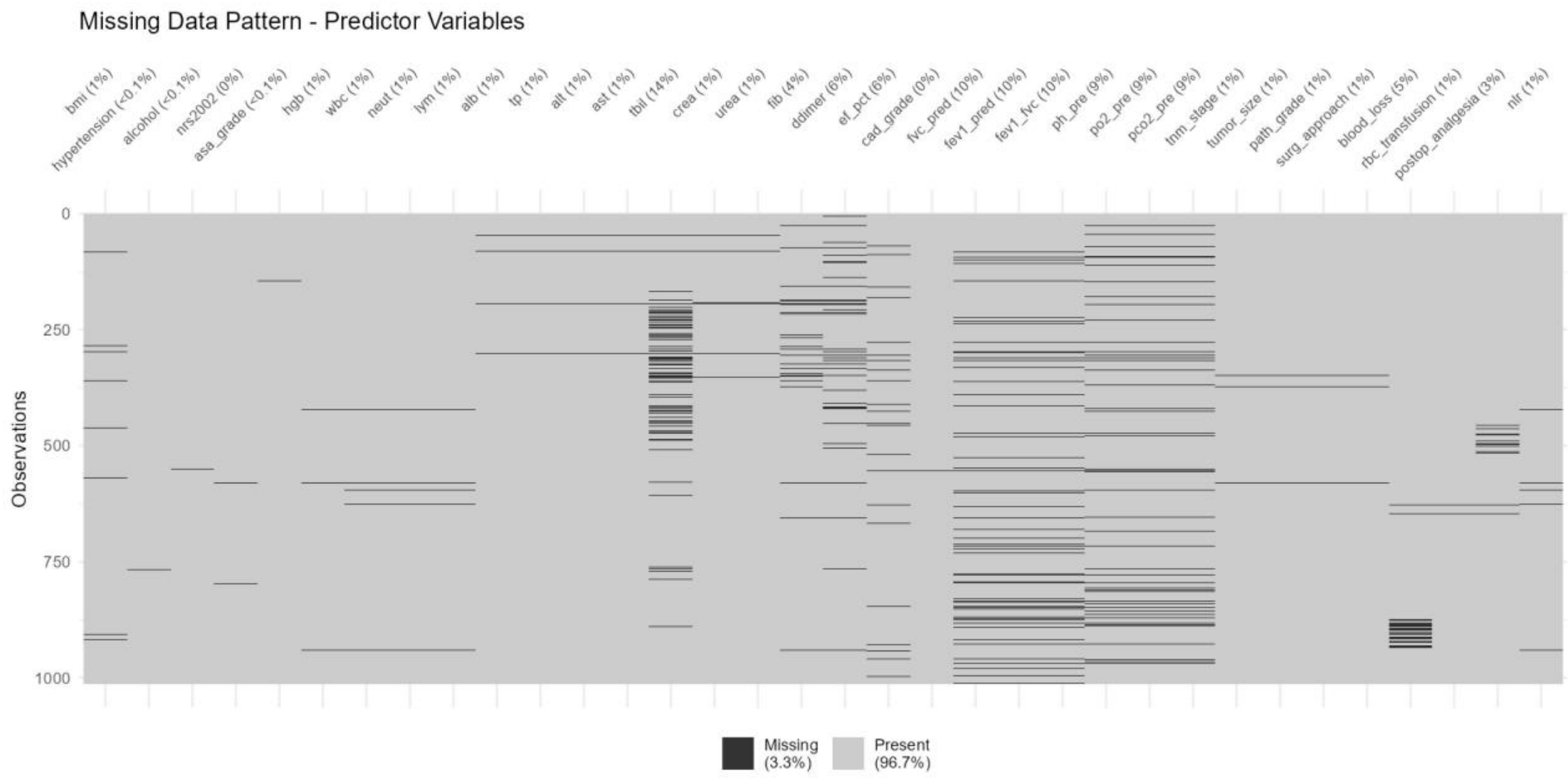

Supplementary Figure S1. Missing data pattern across predictor variables (N = 1013).

Supplementary Figure S1. Missing data pattern across predictor variables (N = 1013). Black bars indicate missing values; gray indicates present data. Overall missingness was 3.3%, predominantly affecting total bilirubin (14%), pulmonary function indices (~10%), and arterial blood gas parameters (~9%). Variables with >1% missingness were handled by MICE (m = 5). MICE, multiple imputation by chained equations.

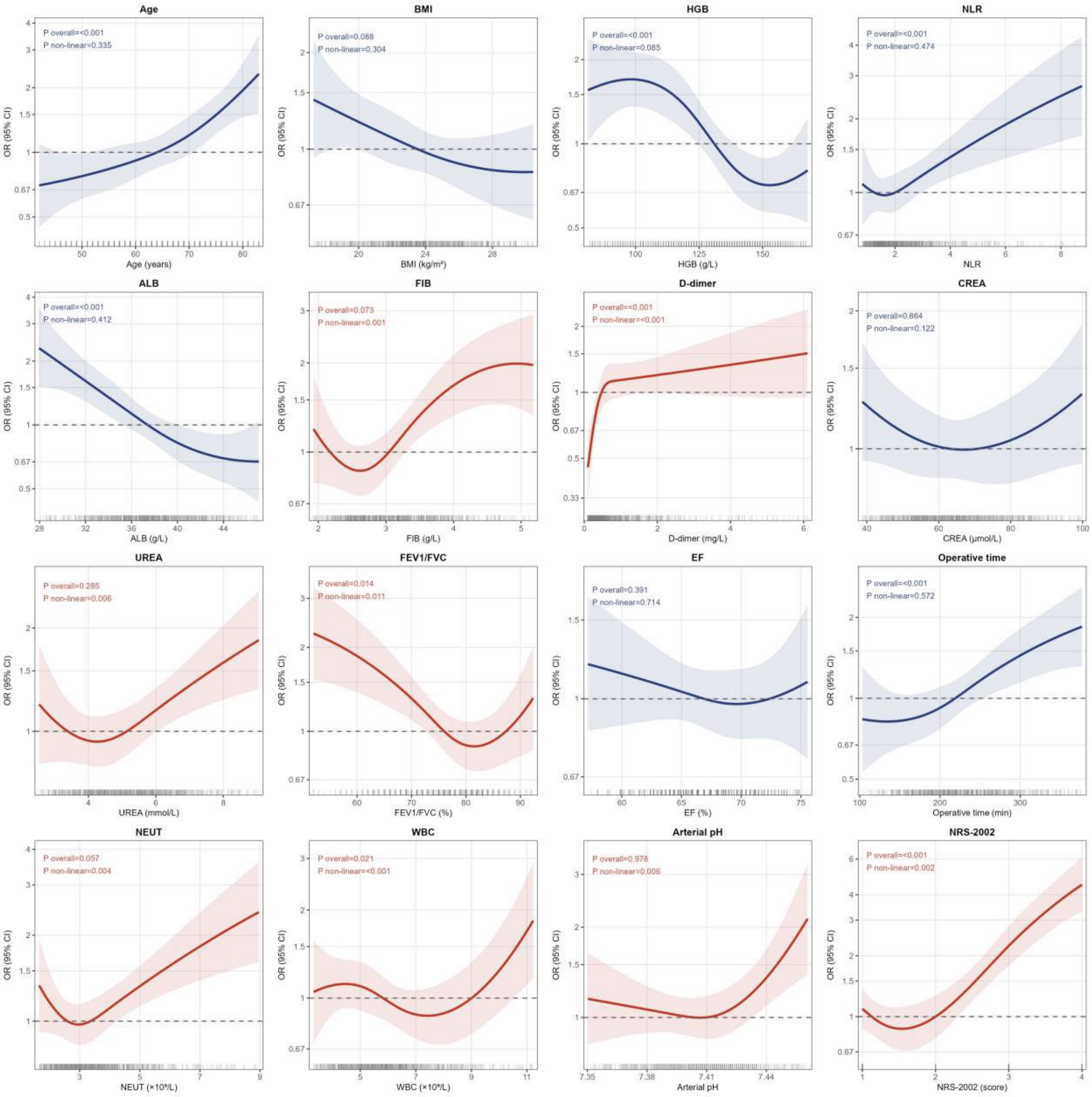

Supplementary Figure S2. Restricted cubic spline analyses of continuous predictors.

Supplementary Figure S2. Restricted cubic spline analyses of continuous predictors.

Odds ratios (solid line) with 95% confidence intervals (shaded band) for each continuous predictor, referenced to its median. P~overall~: overall association; P~non-linearity~: departure from linearity. Variables with P~non-linearity~ < 0.05 were log(1+x)-transformed in subsequent analyses. OR, odds ratio; CI, confidence interval.

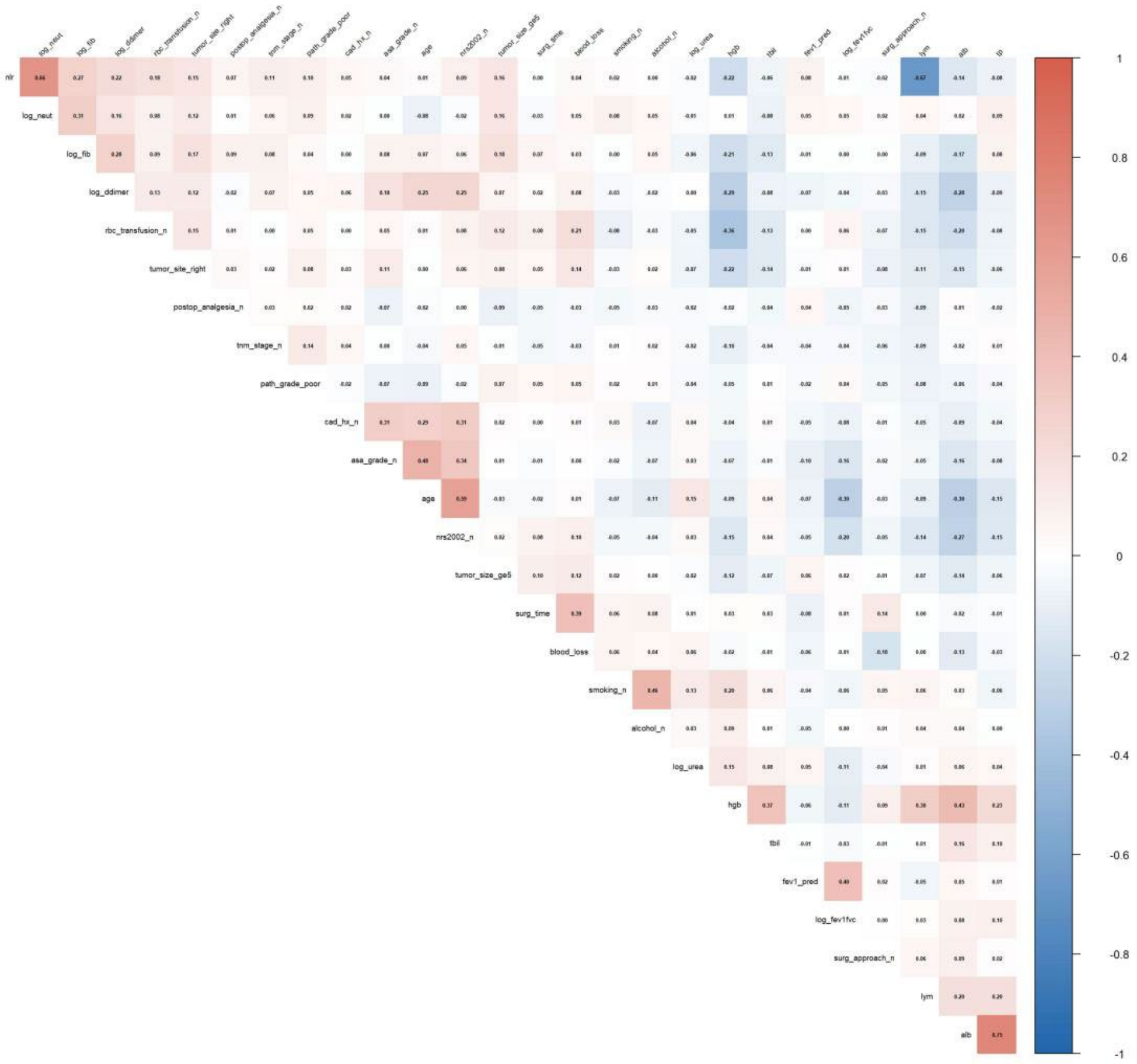

Supplementary Figure S3. Spearman correlation matrix of 27 candidate predictor variables.

Supplementary Figure S3. Spearman correlation matrix of 27 candidate predictor variables.

Color intensity reflects correlation magnitude (red: positive; blue: negative; scale: −1 to +1). All inter-predictor correlations were low (|r| < 0.50), confirming the absence of problematic collinearity. NLR, neutrophil-to-lymphocyte ratio.
