# Supplementary material for: Nutritional risk score drives postoperative morbidity in colorectal cancer: findings from a LASSO-selected multivariable model in 1013 consecutive patients
Source: Front Nutr. 2026 Jul 22;13:1854727. doi: 10.3389/fnut.2026.1854727 (PMC13437264; doi:10.3389/fnut.2026.1854727)
Supplement: Supplementary file 2 [file Table_1.DOCX]

| **Supplementary Table S1A. Prevalence of individual complication systems (N = 1013).** | |
| --- | --- |
| **Complication System** | **Prevalence, n (%)** |
| Hypoalbuminemia | 260 (25.7%) |
| Respiratory complications | 56 (5.5%) |
| Thromboembolic events | 61 (6.0%) |
| Infectious complications | 39 (3.8%) |
| Postoperative nausea/vomiting (PONV) | 43 (4.2%) |
| Cardiac complications | 23 (2.3%) |
| Anastomosis-related complications | 22 (2.2%) |
| Gastrointestinal dysfunction | 23 (2.3%) |
| Hepatorenal insufficiency | 11 (1.1%) |
| *Note:* Values are presented as number and percentage of the total analytic cohort. Thirty-day all-cause mortality: 1.1% (n = 11). | |

| Supplementary Table S1B. Adjusted regression results for recovery outcomes. | | | |
| --- | --- | --- | --- |
| **Outcome** | **Variable** | **GMR/IRR (95% CI)** | **P value** |
| Time to first flatus | Age (years) | 1.001 (0.999-1.003) | 0.419 |
| Time to first flatus | Laparoscopic approach | 0.997 (0.921-1.079) | 0.931 |
| Time to first flatus | Stoma creation | 0.853 (0.809-0.899) | <0.001 |
| Time to first flatus | TNM stage | 0.984 (0.946-1.023) | 0.407 |
| Time to first flatus | Composite complication | 1.080 (1.025-1.138) | 0.004 |
| Time to first stool | Age (years) | 1.000 (0.998-1.002) | 0.815 |
| Time to first stool | Laparoscopic approach | 0.986 (0.916-1.061) | 0.700 |
| Time to first stool | Stoma creation | 0.831 (0.791-0.872) | <0.001 |
| Time to first stool | TNM stage | 0.982 (0.947-1.018) | 0.314 |
| Time to first stool | Composite complication | 1.084 (1.033-1.137) | 0.001 |
| Total hospital stay | Age (years) | 1.000 (0.998-1.002) | 0.822 |
| Total hospital stay | Laparoscopic approach | 0.947 (0.891-1.007) | 0.080 |
| Total hospital stay | Stoma creation | 0.990 (0.950-1.033) | 0.647 |
| Total hospital stay | TNM stage | 1.003 (0.973-1.035) | 0.839 |
| Total hospital stay | Composite complication | 1.248 (1.199-1.300) | <0.001 |
| *Note:* Time to first flatus and time to first stool were analysed by log-linear regression (back-transformed as geometric mean ratio, GMR); total hospital stay was analysed by negative binomial regression (incidence rate ratio, IRR). All models were adjusted for age, surgical approach, stoma creation, TNM stage, and composite complication status. GMR, geometric mean ratio; IRR, incidence rate ratio; CI, confidence interval. | | | |
