# Supplementary material for: Nutritional risk score drives postoperative morbidity in colorectal cancer: findings from a LASSO-selected multivariable model in 1013 consecutive patients
Source: Front Nutr. 2026 Jul 22;13:1854727. doi: 10.3389/fnut.2026.1854727 (PMC13437264; doi:10.3389/fnut.2026.1854727)
Supplement: Supplementary file 3 [file Table_2.DOCX]

| **Supplementary Table S2A. Pre-specified sensitivity analyses.** | | | | | | |
| --- | --- | --- | --- | --- | --- | --- |
| **Analysis** | **N** | **Events, n (%)** | **AUC (apparent)** | **Nagelkerke R²** | **HL test p** | **Notes** |
| Main model (primary analysis) | 1013 | 372 (36.7%) | 0.689 | 0.154 | 0.709 | Reference; 9-system composite; MICE m=5 |
| SA1: Exclude hypoalbuminemia (8-system composite) | 1013 | 183 (18.1%) | 0.688 | 0.118 | 0.867 | Y redefined: 8 systems (hypoalbuminemia excluded); MICE m=5 |
| SA2: Complete case analysis (no imputation) | 813 | 300 (36.9%) | 0.687 | 0.155 | 0.368 | n=813 complete cases; listwise deletion |
| SA3: Laparoscopic subgroup | 899 | 307 (34.1%) | 0.666 | 0.116 | 0.977 | Surg_approach_n dropped (constant); n=899 |
| SA3: Open subgroup (exploratory) | 116 | 65 (56.0%) | 0.803 | 0.346 | — | Exploratory only; n=116 (underpowered) |
| SA4: Base model in TLCO subset | 500 | 178 (35.6%) | 0.715 | 0.193 | 0.574 | Same 5-variable model applied to TLCO subset |
| SA4: Extended model (+TLCO) in TLCO subset | 500 | 178 (35.6%) | 0.715 | 0.193 | 0.638 | TLCO added; DeLong p=0.677 vs base model |
| *Note:*SA1: hypoalbuminaemia excluded from composite endpoint. SA2: complete-case analysis (n = 813). SA3: subgroup analyses by surgical approach (laparoscopic n = 899; open n = 116, exploratory). SA4: base model augmented with TLCO in available subset (n = 500); DeLong's test compared AUCs. AUC, area under the curve; HL, Hosmer–Lemeshow test; TLCO, transfer factor for carbon monoxide. | | | | | | |

| **Supplementary Table S2B. Multivariable model coefficients for the original 9-system composite versus the hypoalbuminaemia-excluded composite (Sensitivity Analysis 1)** | | | | |
| --- | --- | --- | --- | --- |
| **Variable** | **Main model OR (95% CI)** | **Main model P** | **SA1 OR (95% CI)** | **SA1 P** |
| NRS-2002 score | 1.502 (1.312-1.720) | <0.001 | 1.443 (1.232-1.690) | <0.001 |
| Blood loss (mL) | 1.002 (1.001-1.003) | 0.001 | 1.001 (1.000-1.003) | 0.028 |
| Laparoscopic approach | 0.457 (0.297-0.702) | <0.001 | 0.368 (0.233-0.582) | <0.001 |
| Tumor: Right colon | 1.502 (1.044-2.162) | 0.028 | 1.572 (1.040-2.377) | 0.032 |
| NLR | 1.080 (1.015-1.150) | 0.016 | 1.033 (0.974-1.096) | 0.279 |
| Hemoglobin (g/L) | 0.996 (0.989-1.003) | 0.243 | 0.998 (0.990-1.006) | 0.695 |
| Total bilirubin (μmol/L) | 0.977 (0.952-1.002) | 0.080 | 0.997 (0.972-1.023) | 0.825 |
| Operative time (min) | 1.002 (1.000-1.004) | 0.021 | 1.003 (1.001-1.006) | 0.007 |
| *Note*: Both models included the same eight predictors originally selected by LASSO regression in the primary analysis (majority-vote criterion: coefficient non-zero in ≥3 of 5 multiply imputed datasets). Variables were entered simultaneously into multivariable logistic regression and pooled across five multiply imputed datasets using Rubin's rules. Main model: original nine-system composite outcome (N = 1013; events = 372, 36.7%). SA1: composite outcome redefined to exclude hypoalbuminaemia, retaining eight complication systems (N = 1013; events = 183, 18.1%). This comparison was performed to evaluate the stability of model coefficients — particularly for hemoglobin and total bilirubin, which did not reach conventional significance in the main model (P = 0.243 and P = 0.080, respectively) — and to assess whether the original model's predictive performance was specific to hypoalbuminaemia or reflected a broader perioperative risk signal. OR, odds ratio; CI, confidence interval; SA1, Sensitivity Analysis 1; NRS-2002, Nutritional Risk Screening 2002; NLR, neutrophil-to-lymphocyte ratio. | | | | |
