# Supplementary material for: Nutritional risk score drives postoperative morbidity in colorectal cancer: findings from a LASSO-selected multivariable model in 1013 consecutive patients
Source: Front Nutr. 2026 Jul 22;13:1854727. doi: 10.3389/fnut.2026.1854727 (PMC13437264; doi:10.3389/fnut.2026.1854727)
Supplement: Supplementary file 4 [file Table_3.DOCX]

| **Supplementary Table S3. LASSO variable selection across 5 imputed datasets** | | | |
| --- | --- | --- | --- |
| **Variable** | **Separate: IF (lambda.1se, π=0.6)** | **Selected (primary)** | **Stacked (w=1/5)** |
| NRS-2002 score | 5/5 | Yes | Yes |
| Blood loss (mL) | 5/5 | Yes | Yes |
| Laparoscopic approach | 5/5 | Yes | Yes |
| Tumor: Right colon | 5/5 | Yes | Yes |
| NLR | 5/5 | Yes | Yes |
| Hemoglobin (g/L) | 5/5 | Yes | Yes |
| Total bilirubin (μmol/L) | 3/5 | Yes | Yes |
| Operative time (min) | 3/5 | Yes | Yes |
| Postoperative analgesia | 2/5 |  | Yes |
| D-dimer [log] | 1/5 |  | Yes |
| Alcohol use | 1/5 |  | Yes |
| Age (years) | 0/5 |  | Yes |
| Lymphocyte (×10⁹/L) | 0/5 |  |  |
| Albumin (g/L) | 0/5 |  | Yes |
| Total protein (g/L) | 0/5 |  |  |
| FEV₁ (% pred) | 0/5 |  | Yes |
| BUN [log] | 0/5 |  | Yes |
| Fibrinogen [log] | 0/5 |  |  |
| FEV₁/FVC [log] | 0/5 |  | Yes |
| Coronary artery disease | 0/5 |  | Yes |
| Smoking history | 0/5 |  | Yes |
| RBC transfusion | 0/5 |  |  |
| ASA grade | 0/5 |  |  |
| TNM stage | 0/5 |  | Yes |
| Tumor size ≥5cm | 0/5 |  |  |
| Poor differentiation | 0/5 |  | Yes |
| *Note:* Separate Approach (Gunn et al. 2023, Psychol Methods): LASSO fitted independently to each of 5 imputed datasets with 10-fold cross-validated lambda.1se. IF = Inclusion Frequency (number of datasets with non-zero coefficient). pi = 0.6: IF >= 3/5 (primary threshold). Stacked Approach: 5 imputed datasets stacked (weights 1/5); single LASSO with 10-fold CV, lambda.1se. One variable (log-transformed neutrophil count) excluded before LASSO entry due to conceptual redundancy with NLR. | | | |
